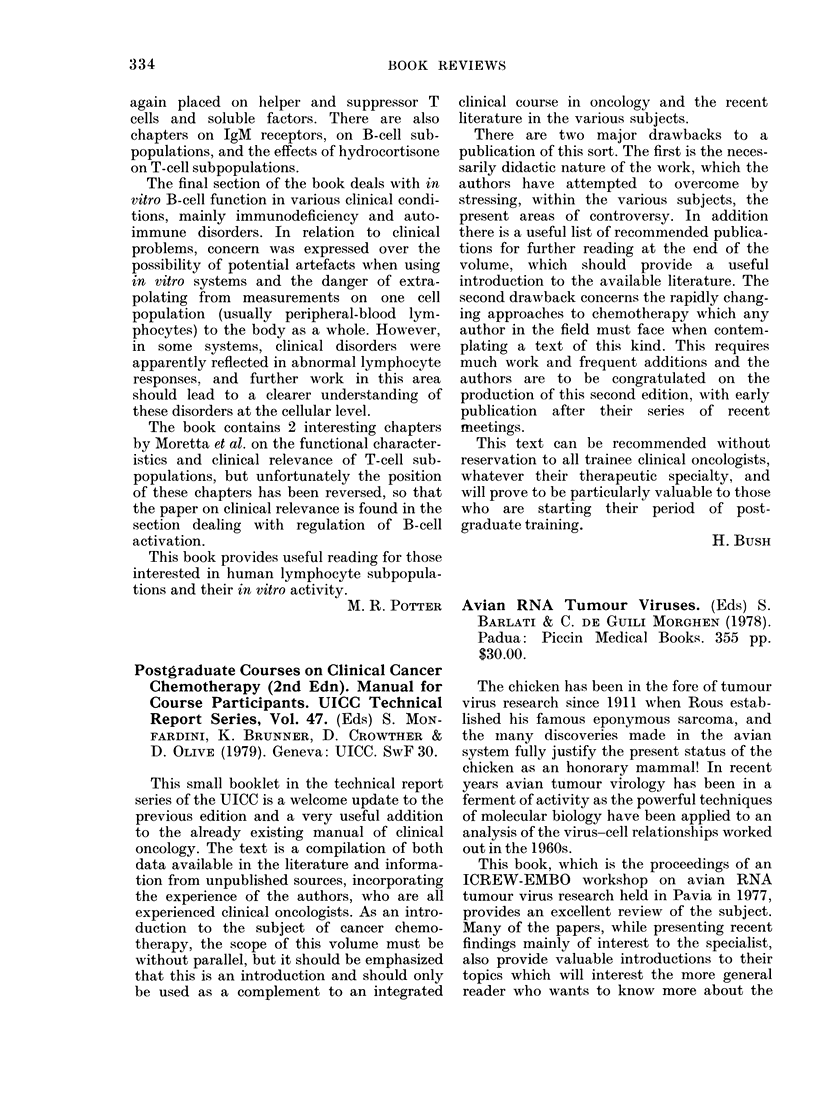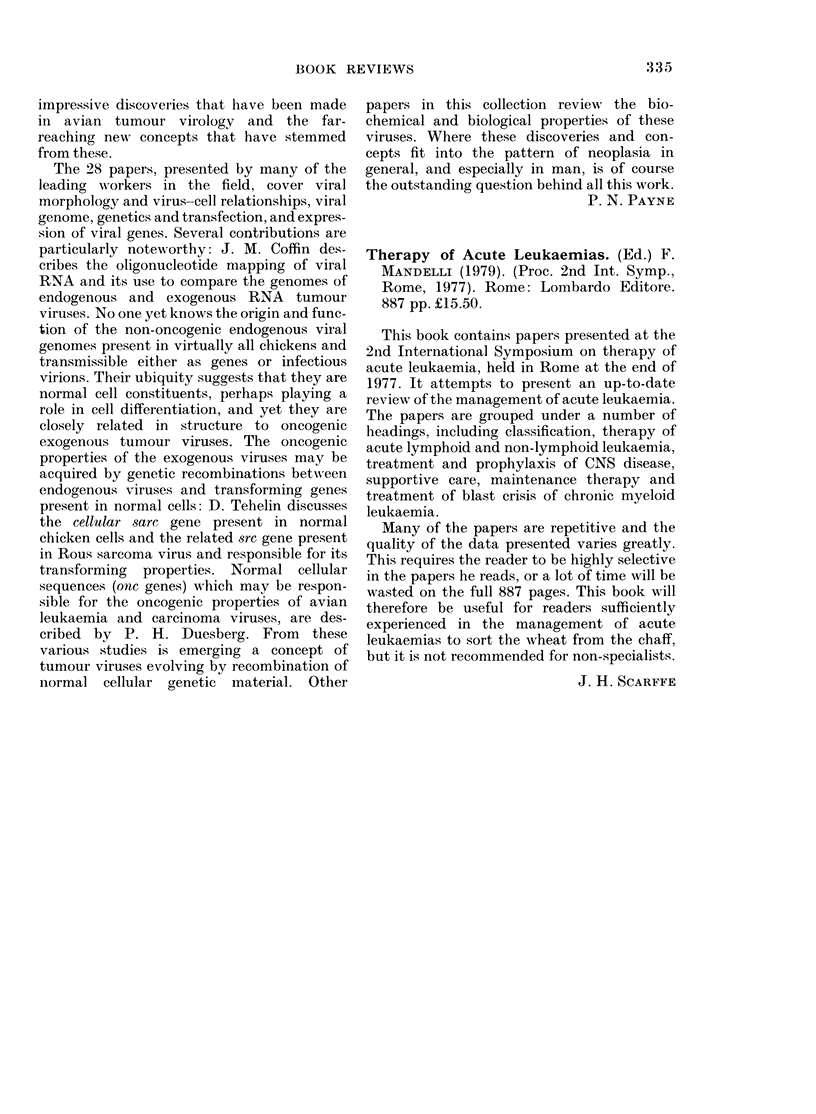# Avian RNA Tumour Viruses

**Published:** 1980-02

**Authors:** P. N. Payne


**Avian RNA Tumour Viruses.** (Eds) S. Barlati & C. de Guili Morghen (1978). Padua: Piccin Medical Books. 355 pp. $30.00.

The chicken has been in the fore of tumour virus research since 1911 when Rous established his famous eponymous sarcoma, and the many discoveries made in the avian system fully justify the present status of the chicken as an honorary mammal! In recent years avian tumour virology has been in a ferment of activity as the powerful techniques of molecular biology have been applied to an analysis of the virus–cell relationships worked out in the 1960s.

This book, which is the proceedings of an ICREW-EMBO workshop on avian RNA tumour virus research held in Pavia in 1977, provides an excellent review of the subject. Many of the papers, while presenting recent findings mainly of interest to the specialist, also provide valuable introductions to their topics which will interest the more general reader who wants to know more about the impressive discoveries that have been made in avian tumour virology and the far-reaching new concepts that have stemmed from these.

The 28 papers, presented by many of the leading workers in the field, cover viral morphology and virus–cell relationships, viral genome, genetics and transfection, and expression of viral genes. Several contributions are particularly noteworthy: J. M. Coffin describes the oligonucleotide mapping of viral RNA and its use to compare the genomes of endogenous and exogenous RNA tumour viruses. No one yet knows the origin and function of the non-oncogenic endogenous viral genomes present in virtually all chickens and transmissible either as genes or infectious virions. Their ubiquity suggests that they are normal cell constituents, perhaps playing a role in cell differentiation, and yet they are closely related in structure to oncogenic exogenous tumour viruses. The oncogenic properties of the exogenous viruses may be acquired by genetic recombinations between endogenous viruses and transforming genes present in normal cells: D. Tehelin discusses the *cellular sarc* gene present in normal chicken cells and the related *src* gene present in Rous sarcoma virus and responsible for its transforming properties. Normal cellular sequences (*onc* genes) which may be responsible for the oncogenic properties of avian leukaemia and carcinoma viruses, are described by P. H. Duesberg. From these various studies is emerging a concept of tumour viruses evolving by recombination of normal cellular genetic material. Other papers in this collection review the biochemical and biological properties of these viruses. Where these discoveries and concepts fit into the pattern of neoplasia in general, and especially in man, is of course the outstanding question behind all this work.

P. N. PAYNE